# Dispersed PM_10_ Microspheres from Coal Fly Ash: Fine Fraction Separation, Characterisation, and Glass–Ceramic Preparation

**DOI:** 10.3390/molecules30122600

**Published:** 2025-06-15

**Authors:** Elena V. Fomenko, Galina V. Akimochkina, Natalia N. Anshits

**Affiliations:** Institute of Chemistry and Chemical Technology, Federal Research Center “Krasnoyarsk Science Center of the Siberian Branch of the Russian Academy of Sciences”, Akademgorodok 50/24, 660036 Krasnoyarsk, Russia; agv3107@mail.ru (G.V.A.); anshitsnn@mail.ru (N.N.A.)

**Keywords:** coal fly ash, aerodynamic classification, magnetic separation, fine narrow fractions, dispersed microspheres, PM_10_, glass–ceramic materials

## Abstract

Developing resource-efficient technologies for producing ceramic materials with specific properties and performance characteristics is one of the most important tasks in modern materials science. As natural resources face depletion, the use of anthropogenic wastes, including fly ash from coal combustion, for the development of new compositions and the production of ceramics with an improved microstructure is of particular significance. The use of PM_10_ fly ash microspheres in ceramic production will help to reduce particulate matter emissions. In this study, fine narrow fractions of PM_10_ microspheres were successfully separated from coal fly ash using aerodynamic and magnetic separation. Glass–ceramic materials with a homogeneous microstructure, an open porosity of 0.4–37%, a compressive strength of 5–159 MPa, and acid resistance of up to 99.9% were obtained using narrow fractions. The materials obtained are promising for application as highly porous ceramics, effective microfiltration membranes, and fine-structured technical ceramics, which can be used in installations operating in aggressive media and/or at high temperatures. The ceramic membranes were characterised by high liquid permeability values up to 1194 L·m^−2^·h^−1^·bar^−1^. Filtration tests showed that the retention coefficient for dispersed microsilica particles with *d*_av_ = 1.9 μm is 0.99.

## 1. Introduction

Thermal power plants (TPPs) generate energy in the process of pulverised coal combustion and produce large volumes of fly ash, thus occupying one of the leading positions among polluters in terms of emissions and the disposal of anthropogenic waste. The main way to reduce the negative impact of TPPs on the environment is to increase the efficiency of solid aerosol particle capture and the level of fly ash utilisation. In the Russian Federation, about 18 million tonnes of coal fly ash (CFA) are generated annually, of which about 30% is recycled. The Russian Energy Strategy-2025 [1] sets a large-scale task of increasing the share of fly ash utilisation up to 40% in 2030, 50% in 2036, and 90% in 2050.

The composition of CFA is heterogeneous and varies considerably; therefore, the utilisation of initial fly ashes without preliminary classification traditionally includes large-tonnage use in the construction industry, agriculture, and road construction. The main methods of CFA utilisation in the world have been published in several review articles [2,3,4,5]. Numerous studies have shown the possibility of obtaining glass–ceramics and ceramics from CFAs with additives of natural raw materials and/or technogenic wastes [6,7,8,9,10,11]. Some data on initial raw materials, including CFA composition and mineral additives; synthesis temperature; and the characteristics of obtained anorthite, wollastonite, cordierite, and mullite ceramics, are presented in Table 1.

Of particular interest, both from an economic and an environmental point of view, is the production of glass–crystalline and glass–ceramic materials from CFA alone without the addition of natural raw materials, nucleating agents, various wastes, or organic binders [12,13,14,15,16,17]. The technological process usually includes the melting of initial ash, hardening, crushing and grinding, screening, pressing, and sintering [12]. DeGuire et al. [13] were among the first to investigate the production, crystallisation, and properties of glasses obtained by melting fly ash of class F without any additives at a high temperature of 1500 °C. Crystalline phases of ferroavgite and potassium melilite were found in the samples. Erol et al. [14] produced glass, glass–ceramic, and ceramic materials from class F fly ash at temperatures of 1500, 1150, and 1125–1200 °C, respectively. The glass–ceramic samples synthesised showed a crystalline augite phase, while the ceramic samples showed enstatite and mullite phases [14]. Mullite ceramics from high-aluminium fly ash without using additional materials but with pre-treatment with alkali and acid to adjust the chemical composition (with the Al_2_O_3_ content reaching 65.35 wt %) were synthesised at 1200–1600 °C [15].

Compared to the methods traditionally used for the production of glass–crystalline and glass–ceramic materials, where the raw material has to be melted at a high temperature to obtain the initial glass [12,13,14,15], direct sintering at a relatively low processing temperature overcomes the high energy consumption, is suitable for mass production, and reduces costs. Usually, direct sintering methods for mineral raw materials lead to the production of fragile samples. It is also not easy to obtain sintered samples from ash alone without any additives and binders. It has been observed [16,17] that the presence of fine particles in CFAs has a positive effect on the properties of the obtained ceramic materials.

Depending on the type and conditions of coal combustion, the fly ash contains up to 40% dispersed particles smaller in size than 10 µm [2,3,18,19]. These particles are anthropogenic atmospheric pollutants, namely, particulate matter (PM_10_), including especially dangerous aerosol particles smaller in size than 2.5 µm (PM_2.5_) [20,21]. The low level of PM_2.5_ and PM_10_ particle capture and utilisation at large coal-fired power plants makes a significant contribution to atmospheric pollution and reduces the quality of the urban environment in large industrial centres.

A significant reduction in the amount of CFA and environmentally hazardous particulate matter can be achieved by processing microspheres with a size of <10 µm and a specific composition, which are potentially suitable for obtaining materials for various purposes. For example, the use of class F fly ash with *d*_av_ = 3 µm [22] and *d*_50_ = 7 µm [23] as an additive in concrete supports an increase in its strength properties. CFA with *d*_50_ = 4 µm [24] was used in the production of fire-resistant boards with significant insulating properties. CFA with *d*_av_ = 4.6 μm was used as a promising filler in polymers [25]. The prospect of using high-calcium CFA with *d*_av_ = 8.5 μm was shown for the production of homogeneous and strong geopolymers [26].

The usefulness of dispersed CFA microspheres in the production of glass–ceramic materials is determined by their composition, including macro-components SiO_2_, Al_2_O_3_, FeO, and CaO; a high content of glass phase; and the morphology and microstructure of powder particles of anthropogenic raw stock. The size of microspheres is a key technological variable in the production of ceramics, which, together with their composition, determines the improved microstructure and properties of the developed composites; obtaining materials from ash waste without additives and binders, without the energy-intensive grinding stage, enables the simplification of the technological process.

Carrying out scientific research to better understand the ecological problems of TPPs related to the emissions and processing of dispersed particles is a very urgent task. The purpose of this research was to separate fractions of dispersed PM_10_ microspheres from CFA, to determine the relationship “composition–structure–properties” for fine narrow fractions, and to form on their basis glass–ceramic materials with an optimal structure and specified indicators depending on the target purpose. The importance of this work is related to the growing demand for new materials that combine efficiency and environmental friendliness and the search for innovative technological solutions for their production. The scientific novelty of this research lies in the development of new approaches to the problem of isolating environmentally hazardous PM_10_ particles from large volumes of waste and the creation of a wide range of materials adapted to specific tasks based on dispersed microspheres from CFA.

## 2. Results and Discussion

### 2.1. Separation of PM_10_ Microspheres from CFA

The potential of dispersed CFA microspheres as a basis for functional and composite materials is determined by the possibility of stabilising their composition and, consequently, properties based on the differences in physical characteristics of individual globules, such as size and magnetic properties [27]. When developing new materials with specified properties, including ceramic composites, on the basis of dispersed microspheres, it is necessary to use homogeneous fractions of a certain composition with a narrow particle size distribution and reproducible physical and chemical characteristics. The possibility of using micro-level homogeneous raw materials in the form of narrow fractions of dispersed microspheres with optimal compositions will help solve the problem of obtaining glass–ceramic materials on the basis of large-tonnage ash wastes, the characteristics of which are comparable with industrially produced technical ceramics.

The involvement of CFA in the economic turnover process depends on the type of coal combustion, which has a significant influence on the chemical composition of the resulting ash [2,3,4,5]. In Russia, most of the coal produced is bituminous (71%), two-thirds of which is used in pulverised combustion processes for energy generation [1]. The combustion of bituminous coal produces aluminosilicate ashes (class F according to ASTM C618 [28]) containing over 70% of the macrocomponents SiO_2_, Al_2_O_3_ and Fe_2_O_3_. The use of this type of ash in the production of glass–ceramics and ceramics has been demonstrated by other researchers [13,14,15], and there is no doubt about its promising potential.

In this study, the characterised fractions of dispersed microspheres were obtained from CFA after the pulverised combustion of bituminous coal from the Kuznetsk Basin, one of the largest coal deposits in the world. This raw material source was chosen due to the large volumes of produced CFA that require utilisation.

**Figure 1:**
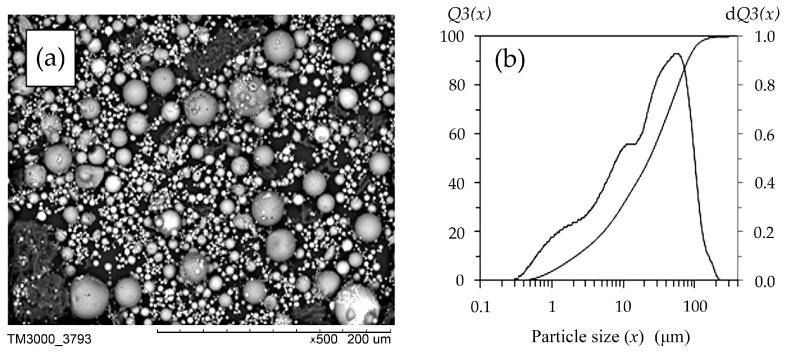
SEM image (**a**) and particle size distribution (**b**) of the original CFA.

**Scheme 1:**
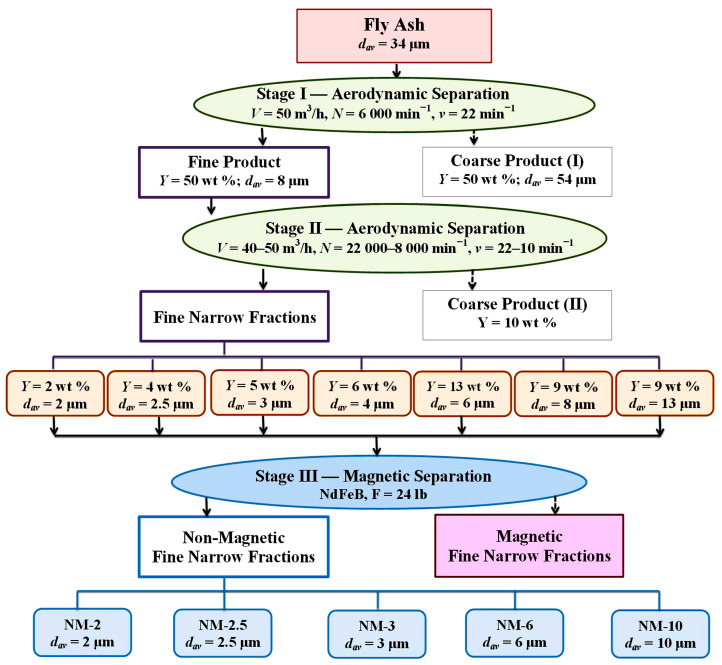
Block diagram showing the technological stages of the separation of narrow fractions of dispersed microspheres from CFA.

The SEM overview image and particle size distribution of the initial ash are presented in Figure 1. The bulk density, magnetic fraction content, particle size distribution characteristics, and chemical and phase compositions of the initial CFA are given in Table 2. The separation of narrow fractions of dispersed microspheres from CFA was carried out according to the technological scheme below (Scheme 1), including the stages of aerodynamic classification under different regimes and magnetic separation. As a result, non-magnetic fine fractions with a narrow particle size distribution and a certain *d*_av_ (indicated by the number in the sample name) were obtained: NM-2, NM-2.5, NM-3, NM-6, and NM-10.

### 2.2. Characterisation of Fine Narrow Fractions

**Figure 2:**
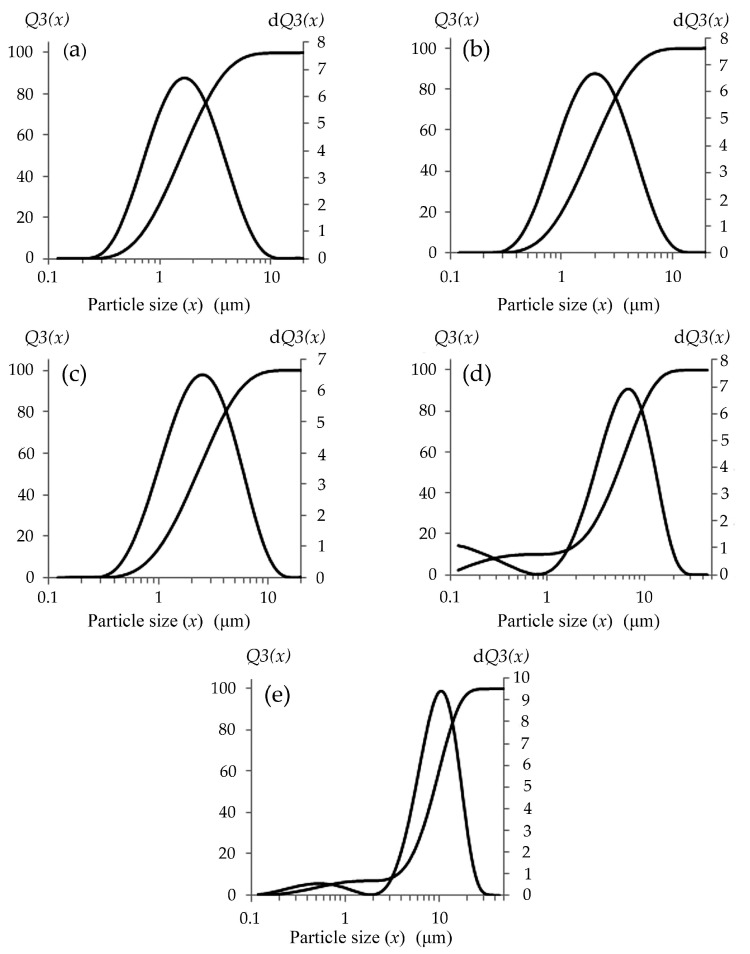
Particle size distribution in cumulative (integral) Q3(x) and differential dQ3(x) forms for narrow fractions of dispersed microspheres: (**a**)—NM-2; (**b**)—NM-2.5; (**c**)—NM-3; (**d**)—NM-6; (**e**)—NM-10.

The bulk density, particle size distribution parameters, and chemical and phase compositions were determined for the fine narrow fractions. The values of bulk density and size distribution characteristics for narrow fractions of dispersed microspheres are given in Table 3. The particle size distributions in cumulative and differential form are shown in Figure 2. The distribution curves show narrow ranges of particle sizes, with *d*_av_ values ranging from 2.1 to 9.9 μm. It was found that with increasing *d*_av_, the bulk density of fractions increases, being in the range of 0.80–1.24 g/cm^3^.

The main components of the chemical composition of the fine narrow fractions (Table 4) are silicon and aluminium oxides: the contents of SiO_2_ and Al_2_O_3_ vary in the range of 59–63 and 25–27 wt%, respectively, and their sum makes 85–88 wt %. The concentration of iron oxide is 5–8 wt. %, which, summed with the aluminosilicate components of the system, reaches 93–94 wt %. According to quantitative XRD analysis, the content of the amorphous glass phase is 91–94 wt % (Table 4). The high proportion of the glass phase is a consequence of the non-equilibrium nature of the pulverised coal combustion process, whereby the thermochemical transformation of mineral forms is incomplete due to high temperature gradients and short contact times [29]. Among the main crystalline phases, mullite and quartz were identified, with a content of 2–4 wt %. The mullite phase is the result of the thermochemical transformation of aluminosilicate minerals present in the original coal, while quartz forms part of its composition [29,30].

**Figure 3:**
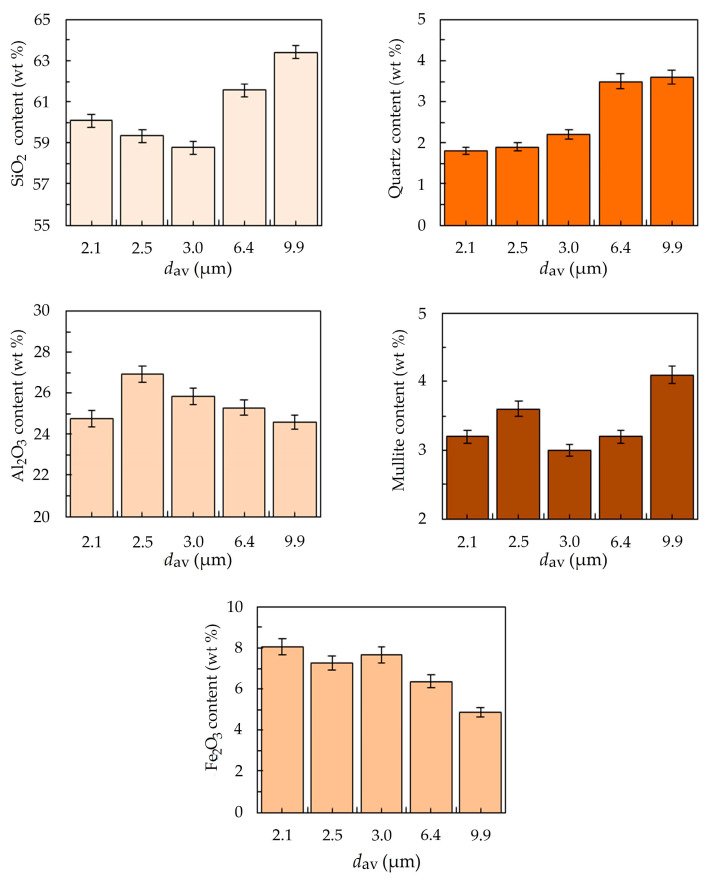
Content of some components in narrow fractions of dispersed microspheres.

The general dependence of the chemical and phase composition of fine narrow fractions on the particle size was also established. The following tendencies were observed: with increasing *d*_av_, the content of SiO_2_ and quartz phase increases, whereas that of Fe_2_O_3_ decreases (Figure 3).

**Figure 4:**
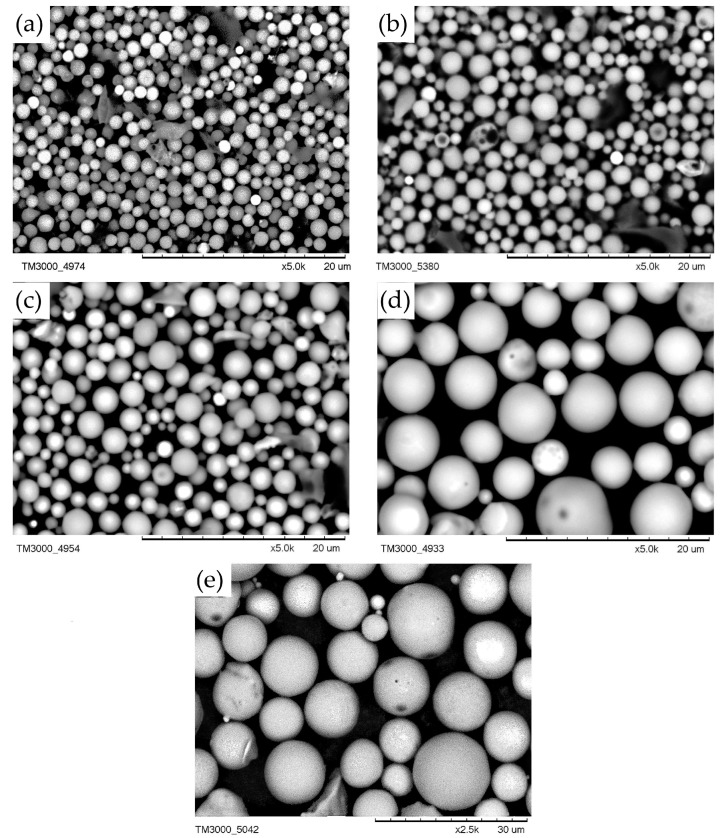
SEM images of narrow fractions of dispersed microspheres: (**a**)—NM-2; (**b**)—NM-2.5; (**c**)—NM-3; (**d**)—NM-6; (**e**)—NM-10.

SEM images of the separated fine narrow fractions (Figure 4) show that they are represented by an absolute majority of spherically shaped particles and are homogeneous in size compared to the original ash (Figure 1). They contain well-fused spherical particles: they are small, with a smooth non-porous shell; with increasing globule size, single pores appear; the number of spheres with a porous shell in the largest fraction is about 15%; splinters are practically absent.

### 2.3. Single-Particle SEM-EDS Analysis

**Figure 5:**
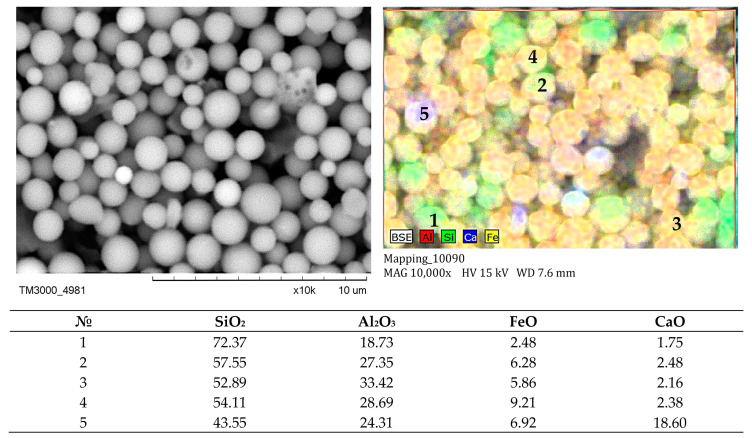
SEM images of narrow fractions of dispersed microspheres NM-2 in elemental mapping mode with indication of compositions of individual globules.

**Figure 6:**
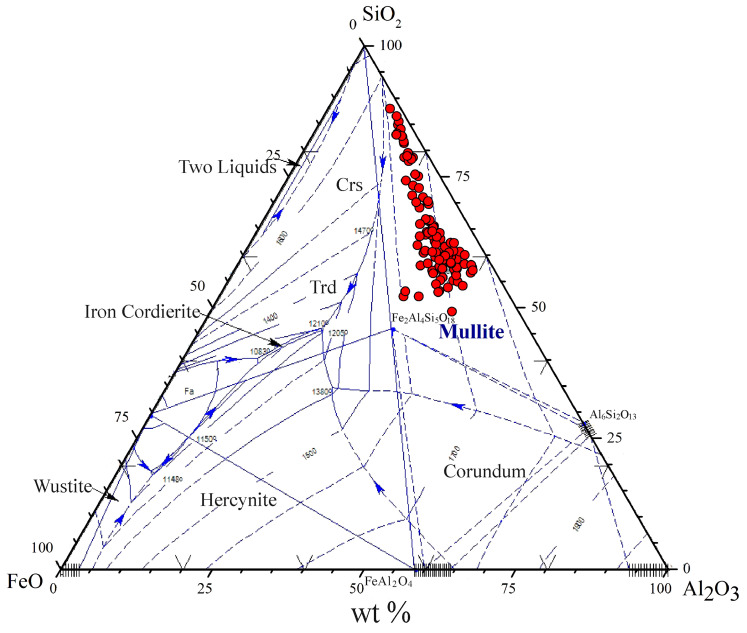
Composition of individual microspheres in ternary diagram coordinates: SiO_2_–Al_2_O_3_–FeO.

A SEM-EDS study of the chemical composition of individual microspheres from 1 to 2 μm in size, part of the PM_2.5_ class of particulate matter posing a specific risk to human health [20,21], was carried out. This size interval also corresponds to the maximum PM_10_ distribution of ash particles from the combustion of different types of coal, which is defined in the literature by the term “fine-fragmentation mode” [31,32]. SEM images in the elemental mapping mode showing the composition of individual microspheres are shown in Figure 5. The main components of individual microspheres are SiO_2_, Al_2_O_3_, FeO, CaO, and MgO, the sum of which exceeds 90 wt % for all microspheres. The analysis of the chemical composition of individual microspheres in the coordinates of the [SiO_2_–Al_2_O_3_–FeO] ternary system [33] (Figure 6) shows that they are located in the regions with primary crystallisation of the mullite phase.

### 2.4. Thermochemical and Phase Transformations in Dispersed Microspheres

The direct sintering of mineral raw materials typically leads to the formation of unstable glass–ceramic samples. It is also difficult to obtain sintered samples containing CFA without pre-classification, with no additives or binders. However, the use of micro-homogeneous raw materials in the form of narrow fractions of dispersed microspheres with an optimal composition has successfully solved the problem of producing glass–ceramic composites [34,35] based on CFA. The properties of these composites are comparable to industrially produced technical ceramics [36].

The performance of glass–ceramic materials is largely determined by their mineral phase composition. A detailed study of the thermochemical and phase transformations occurring in dispersed microspheres during the synthesis of glass–ceramic materials was carried out using simultaneous thermal analysis (DSC-TG) and quantitative X-ray diffraction (XRD).

**Figure 7:**
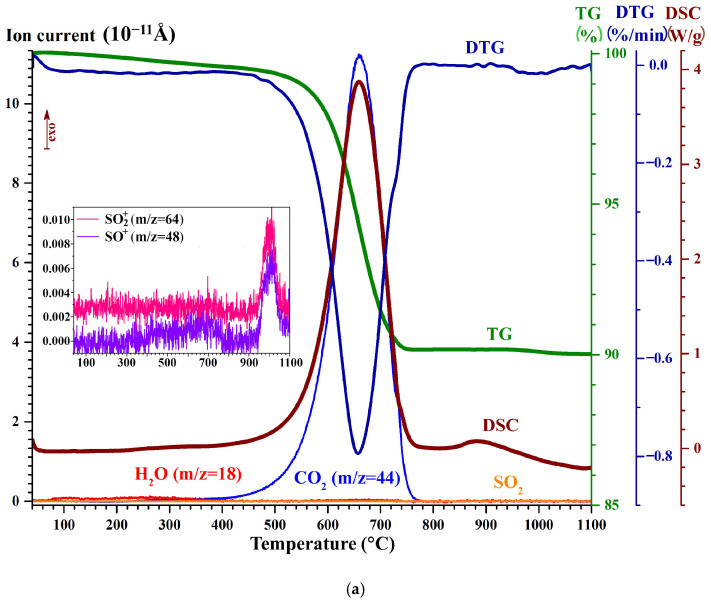

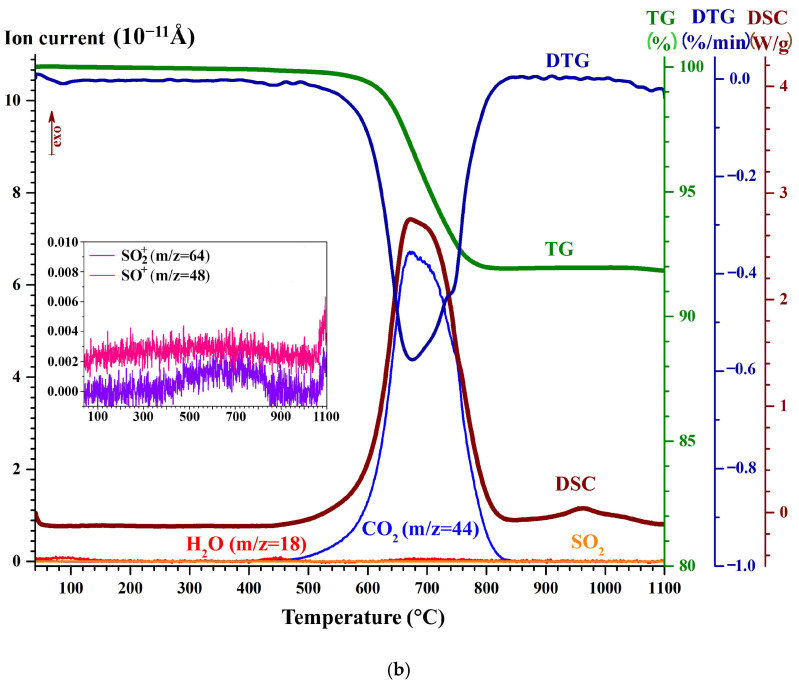
DSC-TG-DTG-MS curves of thermal transformation process for narrow fractions of dispersed microspheres: (**a**)—NM-3; (**b**)—NM-10.

For narrow fractions of dispersed microspheres (NM-3 and NM-10), the following thermochemical transformations were observed in the temperature range of 40–1100 °C (Figure 7). The exothermic peak on the DSC curve at 659 and 672 °C is accompanied by a mass drop and CO_2_ emission, which is caused by the combustion process of coal char (underburning) particles. The observed mass loss in the temperature ranges of 568–742 and 602–797 °C is comparable to the loss on ignition (LOI) value determined via chemical analysis (Table 4).

The crystallisation process of mullite from aluminosilicate glass is observed in the temperature range of 800–950 and 910–986 °C (maximum of 885 and 962 °C for the studied fractions, NM-3 and NM-10). The process of endothermic decomposition of impurity anhydrite begins at temperatures above 995 and 1030 °C for NM-3 and NM-10, respectively.

**Figure 8:**
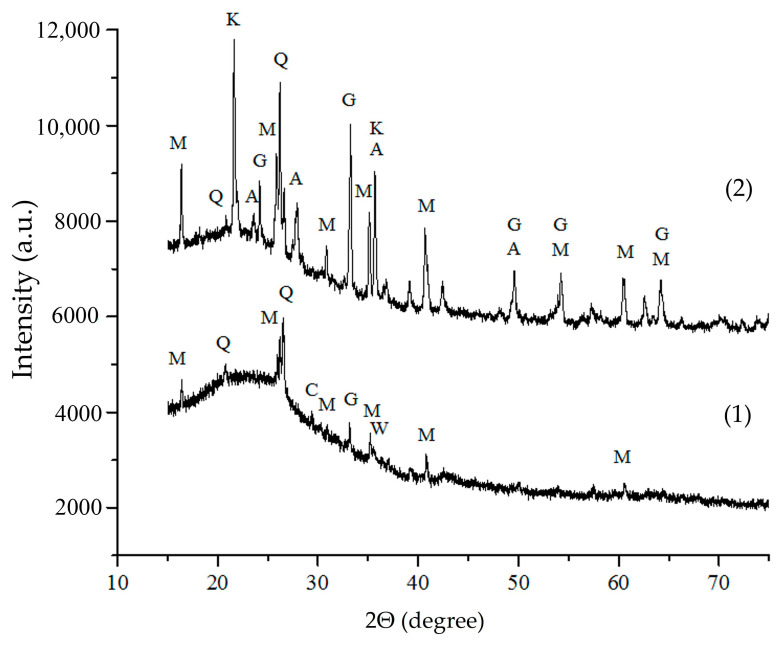
X-ray diffraction patterns of the initial narrow fraction NM-3 (1) and after heat treatment at 1100 °C NM-3/1100 (2): M—mullite (ICDD PDF 01-074-4145), Q—quartz (ICDD PDF 01-070-7344), K—cristobalite (ICDD PDF 01-075-0923), G—hematite (ICDD PDF 01-087-1164), A—anorthite (ICDD PDF 04-011-2883), C—calcite (ICDD PDF 01-086-2334), and W—ferrospinel (ICDD PDF 01-079-0416).

Using quantitative XRD analysis (Figure 8), it was established that, in comparison with the initial narrow fractions of dispersed microspheres (Table 4), crystallisation of the aluminosilicate glass phase occurs during the heat treatment of the samples at 1100 °C in an oxidising atmosphere (Table 5). This crystallisation results in a significant increase in the content of mullite phase from 3–4 to 16–19 wt % and the appearance of cristobalite phase at the level of 2–4 wt %. The anorthite phase is formed at 3–7 wt %. As a result, the amount of glass phase decreases from 91–94 to 67–72 wt %. The amount of quartz decreases, except for the coarse fraction NM-10, where it increases. Reflections of the Fe-spinel and calcite phases, which were characteristic of the initial sample, were not detected.

Thus, during the formation of glass–ceramic materials based on narrow fractions of dispersed microspheres with a macro-component composition of [SiO_2_–Al_2_O_3_–FeO], the following chemical transformations occur, according to Equations (1)–(3):

Al_2_O_3_ (Glass phase) + SiO_2_ (Glass phase) → Mullite Al_6_Si_2_O_13_ − Al_4_SiO_8_ (1)

SiO_2_ (Glass phase) → Cristobalite SiO_2_ (2)

CaCO_3_, CaO (Glass phase) + Al_2_O_3_ (Glass phase) + SiO_2_ (Glass phase) → Anorthite CaAl_2_Si_2_O_8_ (3)

### 2.5. Preparation and Characterisation of Glass–Ceramic Materials

**Figure 9:**
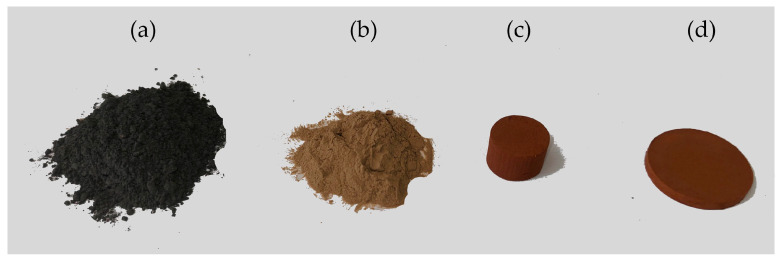
Images of the samples: (**a**) initial fine narrow fraction 2.5 ± 0.2 g used for the preparation of glass–ceramics; (**b**) fine narrow fraction after annealing at 815 °C; (**c**) cylindrical tablet; (**d**) flat membrane.

Glass–ceramic composites based on narrow fractions of dispersed microspheres were obtained by the direct sintering of compacted powder samples [37]. Figure 9 shows the images of the initial CFA fraction and the sample of dispersed microspheres obtained after annealing at 815 °C, producing a glass–ceramic of cylindrical shape and a flat membrane.

The following physicochemical and technical characteristics were determined for glass–crystalline materials according to State Standard indicators [34,35]: sintering coefficient, apparent density, and water absorption [GOST 7025-91]; open porosity [GOST 2409-2014]; compressive strength [GOST R 57606-2017]; and acid resistance [GOST 473.1-2023] [38,39,40,41]. Filtration tests were also carried out on model systems. Table 6 shows the main characteristics of glass–ceramic materials obtained from fractions of dispersed microspheres with *d*_av_ = 3 μm and *d*_av_ = 10 μm. Increasing the sintering temperature significantly increases the apparent density of the samples while reducing their water absorption, open porosity, and pore size. It also enhances their strength and acid resistance.

**Figure 10:**
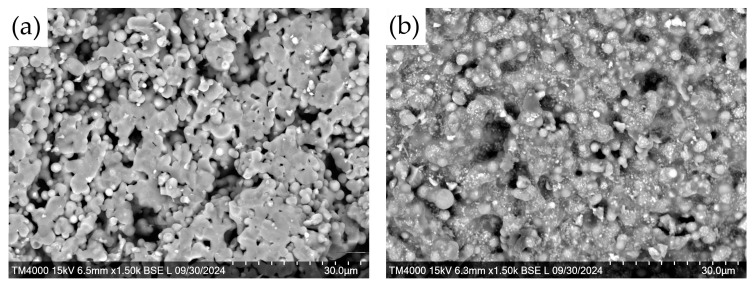
SEM images of the microstructure of glass–ceramics obtained on the basis of the narrow fraction of dispersed NM-3 microspheres at a sintering temperature of (**a**) 1000 and (**b**) 1100 °C.

**Figure 11:**
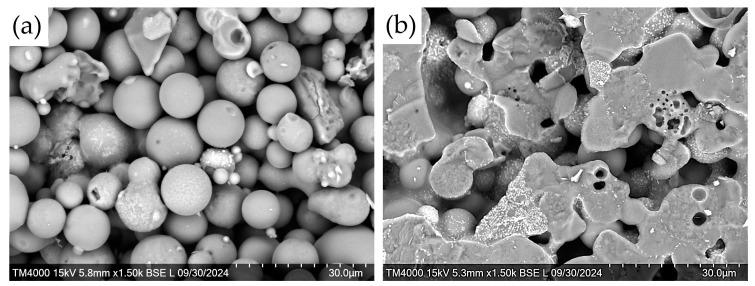
SEM images of the microstructure of glass–ceramics obtained on the basis of the narrow fraction of dispersed NM-10 microspheres at a sintering temperature of (**a**) 1000 and (**b**) 1100 °C.

Narrow fractions of dispersed microspheres, depending on the particle size and ceramic synthesis temperature, are promising for obtaining materials with different degrees of porosity and strong, high-density ceramics (Figure 10 and Figure 11, and Table 6). Regarding the formation of microfiltration membranes (with a required pore size ranging from 0.1 to 10 μm), the samples obtained on the basis of the fine fraction NM-3 at 1000 °C (Figure 10a) and the coarse fraction NM-10 at 1000 °C and 1100 °C (Figure 11) deserve special attention. They are characterised by a combination of open porosity at 18, 37, and 18%; a uniform microporous structure with pore sizes of 0.1–0.7, 0.3–3.6, and 0.1–2.0 µm; and a compressive strength of 48, 5, and 143 MPa, respectively.

**Figure 12:**
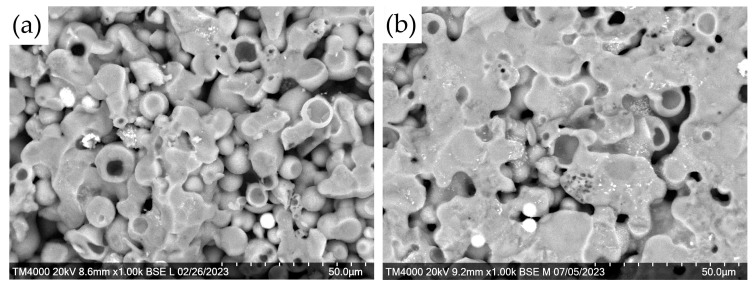
SEM images of the microstructure of glass–ceramics obtained on the basis of the narrow fraction of dispersed HM-10/AR microspheres at a sintering temperature of (**a**) 1100 and (**b**) 1150 °C.

The preliminary acid etching of dispersed microspheres makes it possible to influence the properties of materials. SEM-EDS analysis of the NM-10 fraction showed that for acid-etched globules, iron oxide content decreased by ~2.5 times, magnesium content decreased by ~2 times, and calcium content decreased by ~4 times. This made it possible to increase the sintering temperature of the moulded specimens up to 1150 °C without any noticeable melting while maintaining a porosity of 24% (Figure 12) and increasing the strength up to 159 MPa (Table 6). This helped prevent the leaching of these cations during further processing.

The glass–ceramic materials obtained using narrow fractions of dispersed microspheres from CFA (Table 6) are stronger than those of anorthite, mullite, wollastonite, and cordierite ceramics (Table 1) obtained by other researchers using fly ash with various additives at elevated sintering temperatures [6,7,8,9,10,11]. It is particularly noteworthy that the characteristics of the glass–ceramic materials obtained in this study meet the standard density and porosity parameters (Table 7) for traditional construction ceramics (NM-3/1000 and NM-10/1100) and technical ceramics with a fine structure (NM-10/AR/1150), surpassing them in strength.

The use of semi-permeable membranes is one of the most energy-efficient methods for separating or purifying gases and liquids from micro- and macropollutants [43,44]. Compared to polymeric membranes, ceramic membranes are characterised by higher mechanical strength, chemical and thermal stability, and regenerability, as well as a longer service life [45,46,47,48]. To fully characterise ceramic membranes, in addition to open porosity determination and pore size measurements, filtration tests on model systems with particles of specific sizes are used [35]. Experiments were carried out on the microfiltration of aqueous suspensions of dispersed particles at a pressure drop of 0.6 bar through ceramic membranes synthesised on the basis of narrow fractions of dispersed microspheres. Aqueous suspensions based on microsilica (1 g/L) with the following particle size characteristics were used for filtration experiments: *d*_av_ = 1.9; *d*_10_ = 0.4; *d*_50_ = 1.4; *d*_90_ = 4.2; *d*_99_ = 8.0 μm. The retention coefficient of the dispersed particles was found to be 0.98–0.99 (Table 6). During the filtration process, solid particles were successfully separated. Their deposition occurs on the surface of the ceramic membranes without penetrating into the volume; after mechanical cleaning, the ceramic filters are suitable for reuse. The permeability of ceramic membranes during the purification of aqueous suspensions decreased 2.4–6.5 times compared to the permeability of distilled water (Table 6), which is due to the high concentration of microsilica particles in the filtered aqueous suspension.

The physical and technical characteristics of ceramic membranes obtained in this study (Table 6) are comparable with the parameters of ceramic membranes and substrates obtained in other studies [49,50,51,52,53,54,55,56,57,58,59,60,61,62]. Table 8 presents a comparison of ceramic membranes described in the literature derived from initial fly ashes without prior classification [49,50,51,52,53,54,55,56,57,58,59] with membranes from narrow fractions of dispersed microspheres obtained in this work. The analysis shows that in most cases, ceramic membranes are traditionally synthesised using various additives, such as bauxite [52,53,54,55], kaolin [56,57,58], dolomite [58], mullite [59], etc. In this case, the synthesis temperature is noticeably higher—up to 1550 °C, and the pore size, open porosity, and strength do not show a significant improvement. Ceramic membranes obtained on the basis of clays at sintering temperatures of 950–1000 °C using conventional ceramic technology [60,61,62] are characterised by comparable porosity values of 34–38% and close liquid permeability of 245–725 L·m^−2^·h^−1^·bar^−1^.

Thus, using the method of direct sintering, glass–ceramic materials with homogeneous microstructure, increased strength, and the required porosity and chemical resistance were obtained on the basis of narrow fractions of dispersed CFA microspheres without the grinding of raw materials, additives, or binders. The obtained materials are promising for application as highly porous ceramics, effective microfiltration membranes, and technical ceramics with a fine structure, which can be used in installations operating in aggressive media and/or at high temperatures.

## 3. Materials and Methods

### 3.1. Materials and Sampling

Fly ash of class F according to the ASTM C618 standard [28], obtained from the pulverised combustion of bituminous coal of technological grade T from the Kuznetsk Basin, was used as a raw material for the separation of narrow fractions of dispersed microspheres. Industrial coal combustion was carried out at Moscow TPP-22 (PJSC Mosenergo, Dzerzhinsky, Moscow Region, Russia). CFA sampling was carried out from the first field of electrostatic precipitator (ESP) with a capture efficiency of 99.5–99.8%.

CFA sampling was carried out in accordance with the normative document, which establishes general requirements for representative sampling at thermal power plants [63]. Samples were taken during the period of boiler units’ operation in stable mode at optimum load. According to the regulatory requirements, sampling from ESPs is performed separately from each receiving hopper installed under ESPs in the amount necessary to comprise an average sample of at least 500 kg. A representative sample in the laboratory was obtained according to State Standard GOST [64] when dividing all batches, taking point samples from them and combining and mixing. Regardless of the method of obtaining the sample, it must be thoroughly homogenised.

### 3.2. Separation of Fine Narrow Fractions

Aerodynamic separation was performed on a centrifugal laboratory classifier 50 ATP (Hosokawa ALPINE, Augsburg, Germany) by combining several consecutive classification cycles at different modes. The classifier scheme and the principle of its operation are described in detail in [27]. The technological scheme for the separation of fine narrow fractions from CFA included several stages (Scheme 1) chosen after preliminary tests. In the first stage of aerodynamic classification (Stage I, Scheme 1), 20 kg of original CFA was separated into fine and coarse products with a yield (*Y*) of 50 wt % for each sample. CFA was loaded into the classifier in 2 kg batches (*m*). The air flow rate (*V*) was 50 m^3^/h, the rotor speed (*N*) was 6000 min^−1^, the screw feeder speed (*v*) was 22 min^−1^, and the separation time (*t*) was 15 min.

The fine and coarse products, which were obtained in one stage, significantly differed in terms of particle size, with a *d*_av_ of 8 and 54 μm, respectively. Each of them can be successfully used independently [27] or can be re-classified to obtain narrow fractions with a certain size. In this study, the fine product (Stage I, Scheme 1) was subjected to classification with different classifier modes (Stage II, Scheme 1). The final stage of the technological scheme was wet magnetic separation (Stage III, Scheme 1) performed in distilled water using a neodymium magnet (NdFeB, F—24 lb). Five fine narrow fractions with a *d*_av_ of 2, 2.5, 3, 6, and 10 µm, which were separated at Stage II, were subjected to magnetic separation. After the extraction of the magnetic fractions, we obtained non-magnetic fractions with a narrow particle size distribution and a certain *d*_av_ (denoted by number in the sample name): NM-2, NM-2.5, NM-3, NM-6, and NM-10.

### 3.3. Characterisation Methods

The bulk density, particle size distribution, and chemical and phase compositions were determined for the fine fractions of PM_10_ microspheres.

The bulk density was measured using an automated Autotap density analyser (Quantachrome Instruments, Boynton Beach, FL, USA).

A MicroTec 22 laser analyser (Fritsch GmbH, Idar-Oberstein, Germany) was used to determine the particle size distribution. The measurement was carried out in a wet mode in distilled water using an ultrasonic source to break up the ash particle agglomerates. The globule diameter was determined as the average value of three independent measurements with an absolute error of ±0.3 μm.

The chemical composition of the microspheres was determined using chemical analysis methods according to State Standard GOST 5382-2019 [65]. The precision of analysis is contingent upon the element under scrutiny and the employed method. Table 9 shows the methods and errors of the concentration measurement of each element oxide.

Powder XRD data were collected using a PANalytical X’Pert PRO diffractometer (PANalytical, Almelo, The Netherlands) with a PIXcel detector and graphite monochromator on Cu Kα radiation in the diffraction angular interval of 15–90 2-theta degrees. The crystalline phases were identified using the X’Pert HigScore Plus program version 2.2b (2.2.2) with the ICDD PDF database [66]. The quantitative phase analysis was carried out using the Rietveld method with derivative difference minimisation [67]. The quantity of glass phase was estimated using the external standard method, for which an XRD pattern of purely crystalline corundum was used [68].

An analysis of the composition and morphological structure of the microspheres was carried out by means of scanning electron microscopy (SEM) and X-ray energy-dispersive spectroscopy (EDS) using a TM-3000 scanning electron microscope (High Technologies Corporation, Hitachi, Tokyo, Japan) equipped with an energy-dispersive X-ray spectrometer (Bruker Corporation, Billerica, MA, USA) with the XFlash 430 H detector at the accelerating voltage of 15 kV in the mapping mode with the help of the Quantax 70 system used for X-ray energy-dispersive microanalysis (Bruker Nano GmbH, Berlin, Germany). Powder samples were applied to a double-coated conductive carbon adhesive tape (Ted Pella Inc., Altadena, CA, USA) attached to a flat substrate (1–3 mm thick, 30 mm in diameter) fabricated using Duopur poly(methyl methacrylate) resin (Adler, Schwaz, Austria). Data accumulation time exceeded 10 min, and the quality of the spectrum accumulated in this mode enables quantitative determination of the gross composition of individual globules. The root mean square error of the determination of element content was as follows (%): O—3.0–3.7, Fe—0.7–1.6, Si—0.1–0.6, Al—0.08–0.4, Ca—0.04–0.1, Mg—0.03–0.14, Na—0.03–0.07, K—0.003–0.03, Ti—0.03–0.05, and Mn—0.03–0.05. The concentrations of elements were calculated to determine the content of the corresponding oxides, and iron was used for FeO.

Synchronous thermal analysis was used to study the processes occurring during the heating of dispersed microsphere fractions in the production of glass–ceramic materials. The analysis was conducted using methods such as thermogravimetry, differential scanning calorimetry, and mass spectrometry. The experiments were carried out in a dynamic gas mixture of 20% O_2_ + 80% Ar at a total flow rate of 50 sccm. Changes in mass, heat flux, and gas composition were recorded simultaneously using a Jupiter STA449C thermal analyser equipped with an Aëolos QMS403 mass spectrometer (Netzsch, Selb, Germany). The measurements were taken in platinum–rhodium crucibles without covers at a linear heating rate of 10 °C/min between 40 and 1100 °C. The sample mass was 20 mg. The DSC sensor was calibrated for heat flow by measuring the heat capacity of a sapphire disk in accordance with the method [69]. The licensed NETZSCH Proteus data processing software version 4.8.4 was used for data processing.

### 3.4. Glass–Ceramic Preparation

Prior to the formation of glass–ceramic materials to stabilise the chemical composition, narrow fractions of dispersed microspheres were subjected to annealing to remove unburned carbon particles present in CFA, and individual samples were subjected to acid treatment to remove leachable cations.

To remove unburned carbon particles, annealing was carried out at 815 °C (oxidising atmosphere, 1 h). Acid treatment was performed in a mixture of concentrated nitric acid and hydrochloric acid (HNO3/HCl = 1:3, lat. Aqua Regia). The ratio of dispersed microspheres to acid was 1:3. The mixture was stirred constantly for two hours, after which it was washed with distilled water until the pH was neutral and dried at 115 °C until it reached a constant weight.

Glass–ceramic composites based on narrow fractions of dispersed microspheres were obtained by compacting powder samples via cold static uniaxial pressing in a closed rigid mould [37] on a Carver #4350 laboratory hydraulic press (Carver Inc., Wabash, IN, USA), followed by high-temperature firing in a muffle furnace.

Dry pressing was used to obtain glass–ceramics from the narrow ash fraction with *d*_av_ = 3 μm, and for the narrow ash fraction with *d*_av_ = 10 μm, 10 wt % distilled water was added. Cylindrical tablets with a diameter of 10–16 mm and a height of 8–15 mm were obtained at pressures of 50 and 100 MPa. Flat membranes with a diameter of 28 mm and a thickness of 4 mm were obtained at a pressure of 40 MPa.

Prior to muffle sintering, the pressed samples were dried at 90–105 °C for 1–2 h to remove moisture to a constant mass. Sintering in the muffle furnace was carried out in the temperature range of 1000 to 1150 °C with a holding time of 2–3 h. The temperature regime was chosen according to the results of the thermal analysis.

### 3.5. Glass–Ceramic Characterisation Techniques

For the glass–ceramic materials, the following characteristics were determined [34,35]: sintering coefficient, apparent density, and water absorption [38]; open porosity [39]; compressive strength [40]; and acid resistance [41].

The morphology of glass–ceramic materials was studied using a TM-4000 scanning electron microscope (High Technologies Corporation, Hitachi, Tokyo, Japan) in the back-reflected electron mode at accelerating voltages of 15 kV and 20 kV.

The porous structure of the samples was investigated using a Porolux 1000 capillary flow porometer (Aptco Technologies NV, Nazareth, Belgium) [35].

The permeability and filtration properties of ceramic membranes were studied using a laboratory vacuum unit. An aqueous suspension of microsilica (*d*_av_ = 1.9; *d*_10_ = 0.4; *d*_50_ = 1.4; *d*_90_ = 4.2; *d*_99_ = 8.0 μm; 1 g/L) was fed at atmospheric pressure to the membrane, at the back of which a pressure drop of 0.6 bar was created using a water jet pump. The concentration of particles in the stock solution and permeate was determined using the photometric method using a Genesys 10S UV–Visible spectrophotometer (Thermo Fisher Scientific, Waltham, MA, USA). Optical density was measured at a wavelength of 540 nm in cuvettes with an optical path length of 50 mm. The retention coefficient, which characterises the filtration efficiency, was calculated according to Equation (4):

k = 1 − C_p/_C_f_, (4)

where C_p_ is the particle concentration in the permeate (mg/L), and C_f_ is the particle concentration in the feed solution (mg/L). Membrane permeability was determined for distilled water and aqueous suspension as the ratio of the amount of permeate to the product of the membrane area and process time.

## 4. Conclusions

Fine narrow fractions of PM_10_ microspheres with an average diameter *d*_av_ of 2, 2.5, 3, 6, and 10 µm were successfully separated from CFA using a combination of aerodynamic and magnetic separation techniques. The separated fractions of the dispersed microspheres had a bulk density ranging from 0.80 to 1.24 g/cm^3^. The chemical composition was mainly represented by SiO_2_ and Al_2_O_3_ at 59–63 and 25–27 wt %, respectively. The phase composition includes an amorphous glass phase (91–94 wt %), as well as crystalline phases of mullite and quartz (3–4 and 2–4 wt %, respectively).

A detailed study of the thermochemical and phase transformations that occur in PM_10_ microspheres during the synthesis of glass–ceramic materials was conducted. It was established that the crystallisation of the glass phase leads to an increase in the mullite phase content of up to 19 wt %, as well as the formation of cristobalite and anorthite phases in amounts up to 4 and 7 wt %, respectively. The amount of the glass phase decreased by up to 67 wt %.

Glass–ceramic materials with an apparent density of 1.4–2.8 g/cm^3^, an open porosity of 0.4–37%, a compressive strength of 5–159 MPa, and acid resistance up to 99.9% were obtained on the basis of narrow fractions of dispersed PM_10_ microspheres using the direct sintering method. These materials are promising for the creation of technical ceramics and microfiltration membranes. The resulting ceramic membranes were characterised by high liquid permeability values of up to 1194 L·m^−2^·h^−1^·bar^−1^. Filtration testing showed that the retention coefficient of dispersed microsilica particles with *d*_av_ = 1.9 μm was 0.99, and acid resistance was 96–99%. Ceramic filters can be reused after mechanical cleaning. Thus, the regularities of the process of obtaining glass–ceramic materials with a set of specified properties on the basis of dispersed PM_10_ microspheres separated from CFA were established.

The results of this study form the basis for developing new science-intensive technologies for processing large quantities of waste from thermal power engineering into improved materials with specified properties and performance characteristics.

## Figures and Tables

**Table 1:** Characteristics of fly ash-based glass–ceramic materials.

Glass–Ceramic Materials	Raw Materials		Temperature (°C)	Characteristics of Glass–Ceramic Materials	Reference
	Fly Ash Composition (wt %)	Additives			
Anorthite porous ceramics	SiO_2_—54.08, Al_2_O_3_—38.00, Fe_2_O_3_—1.89, CaO—1.08	Steel slag	1120–1200	Apparent porosity: 5–65% Flexural strength: 5–60 MPa	[6]
Wollastonite glass–ceramic	SiO_2_—43.24, Al_2_O_3_—20.49, Fe_2_O_3_—4.99, CaO—24.38	Glass	850–1050	Density: 2.042–2.501 g/cm^3^. Flexural strength: 60.8–94.1 MPa Compressive strength: 162.7–238.6 MPa	[7]
Wollastonite glass–ceramic	SiO_2_—55.30, Al_2_O_3_—29.36, Fe_2_O_3_—5.84, CaO—4.58	Na_2_O CaCO_3_	1550	Density: 2.58–2.72 g/cm^3^ Four-point flexural strength: 48–103 MPa	[8]
Cordierite glass–ceramic	SiO_2_—57.99, Al_2_O_3_—28.98, Fe_2_O_3_—4.33, CaO—2.49	Al_2_O_3_ MgCO_3_	1125–1320	Density: 1.57–2.05 g/cm^3^ Compressive strength: 35–50 MPa	[9]
Mullite ceramic	SiO_2_—50.70, Al_2_O_3_—36.38, Fe_2_O_3_—5.63, CaO—3.59	Al_2_O_3_	1600	Porosity: 77–79%. Frame density: 2.59–2.89 g/cm^3^ Compressive strength: 1.21–2.33 MPa	[10]
Mullite ceramic	SiO_2_—44.3, Al_2_O_3_—47.9, Fe_2_O_3_—1.73, CaO—2.11	Al_2_O_3_ AlF_3_	1200	Porosity: 20–28%. Density: 0.6–1.3 g/cm^3^ Flexural strength: 24–59 MPa	[11]

**Table 2:** Bulk density, magnetic fraction content, particle size distribution parameters, and chemical and phase compositions of the original CFA.

Bulk Density (g/cm^3^)	Magnetic Fraction Content (wt %)	Particle Size Distribution (µm)						Phase Composition (wt %)							
		*d*_av_	*d*_10_		*d*_50_		*d*_90_	Glass Phase	Quartz		Mullite		Fe-Spinel		Hematite
1.39	7.9	34	2		23		80	86.8	5.2		2.9		3.9		1.2
Chemical composition (wt %) *															
LOI **	SiO_2_	Al_2_O_3_		Fe_2_O_3_		CaO		MgO		Na_2_O		K_2_O		SO_3_	
10.70	60.52	26.30		6.62		3.28		1.24		0.62		1.35		0.07	

* Contents of silicon, aluminium, iron, calcium, magnesium, sodium, potassium, and sulphur oxide were recalculated: the LOI value was subtracted from the result of the bulk chemical analysis, and the sum of oxides was reduced to 100%. ** LOI—loss on ignition.

**Table 3:** Bulk density and particle size distribution characteristics for narrow fractions of dispersed microspheres.

Fraction	Bulk Density (g/cm^3^)	Particle Size Distribution (µm)				
		*d*_av_	*d*_10_	*d*_50_	*d*_90_	*d*_99_
NM-2	0.80	2.1	0.7	1.7	4.1	7.3
NM-2.5	1.05	2.5	0.8	2.0	4.8	8.3
NM-3	1.14	3.0	0.9	2.4	5.8	10.0
NM-6	1.23	6.4	0.9	5.6	12.4	19.5
NM-10	1.24	9.9	3.8	9.4	16.9	24.2

**Table 4:** Chemical and phase compositions of narrow fractions of dispersed microspheres.

Fraction	Chemical Composition (wt %) *									Phase Composition (wt %)					
	LOI	SiO_2_	Al_2_O_3_	Fe_2_O_3_	CaO	MgO	Na_2_O	K_2_O	SO_3_	Glass Phase	Mullite	Quartz	Fe-Spinel	Hematite	Calcite
NM-2	16.20	60.07	24.76	8.03	3.30	1.04	0.74	1.95	0.11	94.1	3.2	1.8	0.5	0.4	–
NM-2.5	12.28	59.33	26.93	7.26	2.98	0.87	0.69	1.83	0.11	93.4	3.6	1.9	0.9	0.2	–
NM-3	9.85	58.76	25.85	7.66	4.61	1.32	0.67	1.06	0.07	93.1	3.0	2.2	0.9	0.5	0.3
NM-6	7.60	61.55	25.29	6.36	3.15	1.19	0.67	1.70	0.09	92.3	3.2	3.5	–	0.7	0.3
NM-10	7.30	63.41	24.59	4.88	3.02	1.53	0.70	1.79	0.08	91.4	4.1	3.6	–	0.6	0.3

* Contents of silicon, aluminium, iron, calcium, magnesium, sodium, potassium, and sulphur oxide were recalculated: the LOI value is subtracted from the result of the bulk chemical analysis, and the sum of oxides is reduced to 100%.

**Table 5:** Phase composition (wt %) of narrow fractions of dispersed microspheres after heat treatment at 1100 °C.

Fraction	Glass Phase	Mullite	Quartz	Hematite	Cristobalite	Anorthite
HM-2/1100	70.4	18.5	0.7	3.6	3.5	3.3
HM-2.5/1100	71.8	16.2	0.8	3.5	2.9	4.8
HM-3/1100	69.5	16.5	1.3	3.7	3.4	5.6
HM-6/1100	67.0	16.8	3.1	2.7	3.4	7.0
HM-10/1100	66.8	18.7	5.6	1.3	2.3	5.3

**Table 6:** Characteristics of glass–ceramic materials based on narrow fractions of dispersed microspheres.

Parameter	NM-3		NN-10		NM-10/AR *	
Sintering temperature (°C)	1000	1100	1000	1100	1100	1150
Sintering coefficient	0.61	0.44	0.90	0.64	0.68	0.64
Apparent density (g/cm^3^)	1.78	2.76	1.35	1.82	1.63	1.89
Water absorption (%)	10	0.2	28	10	19	14
Open porosity (%)	18	0.4	37	18	31	24
Compressive strength (MPa)	48	56	5	143	99	159
Minimum pore size (µm)	0.10	–	0.28	0.07	0.32	0.18
Average pore size (µm)	0.12	–	0.92	0.22	0.92	0.34
Maximum pore size (µm)	0.67	–	3.60	2.03	2.71	1.12
Acid resistance (%)	97.3	98.9	96.3	98.7	99.9	99.9
Water permeability (L·m^−2^·h^−1^·bar^−1^)	24	–	1194	217	240	170
ASMS ** permeability (L·m^−2^·h^−1^·bar^−1^)	10	–	265	82	–	76
Retention coefficient	0.99	–	0.99	0.99	0.99	0.99

* AR—lat. Aqua Regia (a narrow fraction of dispersed microspheres was subjected to acid etching). ** ASMS—aqueous suspension of microsilica.

**Table 7:** Comparison of the performance of conventional technical ceramics [42] and the dispersed microsphere glass–ceramics obtained in this study.

Ceramics	Apparent Density (g/cm^3^)	Open Porosity (%)	Compressive Strength (MPa)
Building bricks	1.6–1.9	15–20	7.5–20
Faience	1.9–2.4	18–20	100
Narrow fractions of dispersed microspheres (this work)	1.8–1.9	18–24	48–159

**Table 8:** Comparison of the performance of fly ash-based ceramic membranes reported in the literature [49,50,51,52,53,54,55,56,57,58,59] with the dispersed microsphere membranes obtained in this study.

Membrane Material	Sintering Temperature (°C)	Pore Size (µm)	Open Porosity (%)	Flexural Strength (MPa)	Reference
Fly ash	1200	0.7–3.0	–	–	[49]
Fly ash + Al(OH)_3_ + MoO_3_	1200–1500	0.2–1.6	30–58	26–141	[50]
Fly ash + CaCO_3_	1200–1350	0.6–1.7	0–50	34–90	[51]
Fly ash + bauxite + AlF_3_ + MoO_3_	1100–1500	0.3–0.5	10–50	20–150	[52]
Fly ash + bauxite + WO_3_	1200–1400	0.7–1.8	45–52	35–88	[53]
Fly ash + bauxite +TiO_2_	1300–1500	5–10	39–47	27–36	[54]
Fly ash + bauxite	1150–1300	2–4	0–40	20–95	[55]
High-alumina fly ash + kaolin + alumina + graphite	1150–1550	–	40–70	–	[56]
Fly ash + kaolin + CaCO_3_, Na_2_CO_3_, H_3_BO_3_	750–900	0.3–2	30–40	7–26	[57]
Fly ash + kaolin + dolomite	800–1000	0.4–2.5	35–50	15–55	[58]
Fly ash + mullite	1000–1200	1–2	30–35	15–50	[59]
Narrow fractions of dispersed microspheres with *d*_av_ = 10 μm	1000–1150	0.1–3.6	18–37	5–159 *	This work

* Compressive strength.

**Table 9:** Chemical analysis methods and measurement errors for the concentration of each oxide element according to [65].

Oxide	Method	The Mean Squared Error	The Discrepancy Between Two Parallel Definitions
SiO_2_	Gravimetric analysis	±0.02 ÷ ±0.35	0.03 ÷ 0.50
Al_2_O_3_	Complexometric titration	±0.05 ÷ ±0.30	0.07 ÷ 0.40
Fe_2_O_3_	Complexometric titration	±0.15 ÷ ±0.60	0.20 ÷ 0.80
FeO	Permanganometric titration	±0.02 ÷ ±0.04	0.03 ÷ 0.05
CaO	Complexometric titration	±0.15	0.20
MgO	Complexometric titration	±0.10 ÷ ±0.20	0.15 ÷ 0.30
Na_2_O	Flame photometric method	±0.04 ÷ ±0.10	0.06 ÷ 0.15
MnO	Photocolorimetry	±0.04	0.05
SO_3_	Gravimetric analysis	±0.04	0.06

## Data Availability

The data presented in this study are available upon request from the corresponding author.

## Abbreviations

The following abbreviations are used in this manuscript:

ASMS	Aqueous suspension of microsilica.
AR	lat. Aqua Regia.
CFA	Coal fly ash.
DSC-TG	Differential Scanning Calorimeter—Thermogravimetry.
EDS	Energy-dispersive spectroscopy.
ESP	Electrostatic precipitator.
LOI	Loss on ignition.
PM	Particulate matter.
SEM	Scanning electronic microscopy.
TPPs	Thermal power plants.
XRD	X-ray diffraction.
